# The Role of Cannabis within an Emerging Perspective on Schizophrenia

**DOI:** 10.3390/medicines5030086

**Published:** 2018-08-08

**Authors:** Jegason P. Diviant, Jacob M. Vigil, Sarah S. Stith

**Affiliations:** 1 Department of Psychology, University of New Mexico, Albuquerque, NM 87131, USA; diviantj@unm.edu; 2 Department of Economics, University of New Mexico, Albuquerque, NM 87131, USA; ssstith@unm.edu

**Keywords:** schizophrenia, cannabis, marijuana, autoimmunity, monoamine therapy, mental illness, cannabidiol, tetrahydrocannabinol, endocannabinoid system

## Abstract

**Background:** Approximately 0.5% of the population is diagnosed with some form of schizophrenia, under the prevailing view that the pathology is best treated using pharmaceutical medications that act on monoamine receptors. **Methods:** We briefly review evidence on the impact of environmental forces, particularly the effect of autoimmune activity, in the expression of schizophrenic profiles and the role of *Cannabis* therapy for regulating immunological functioning. **Results:** A review of the literature shows that phytocannabinoid consumption may be a safe and effective treatment option for schizophrenia as a primary or adjunctive therapy. **Conclusions:** Emerging research suggests that *Cannabis* can be used as a treatment for schizophrenia within a broader etiological perspective that focuses on environmental, autoimmune, and neuroinflammatory causes of the disorder, offering a fresh start and newfound hope for those suffering from this debilitating and poorly understood disease.

Schizophrenia is arguably among the most severe, costly, and mechanistically complex mental illnesses, and yet is relatively common, affecting roughly 0.5% of the US population. [1,2,3,4]. Historical theories of the etiology of schizophrenia have changed over time, and with them the types of interventions conventionally used for treating people with schizophrenic-like, i.e., positive and negative, symptoms. Currently, genetic and epigenetic vulnerability models remain the prevailing dogma, whereby schizophrenic symptoms are believed to manifest from an aberrant or sensitive underlying genotype, independent of or in coincidence with exposure to an environmental (biological or social) risk factor at some point in early development [5,6,7].

The genotypic-centered perspective has mostly been coupled with the assumption that the primary locations of health disturbances are pathophysiological perturbations in neurotransmission or the regulation of brain chemicals [8,9]. Antipsychotic medications frequently prescribed to treat schizophrenia are often designed around a monoamine neurotransmitter hypothesis, typically the dopamine hypothesis, which associates the disorder with a dysfunction in the dopaminergic pathways, contributing to positive, negative, and cognitive symptoms of the disease [10,11,12]. First-generation (typical) antipsychotics share the primary pharmacological property of D2 antagonism. The postulate is that a hyperactive mesolimbic pathway may cause positive psychotic symptoms. The desired efficacy of typical antipsychotics is achieved by blocking 60–65% of D2 receptors in the mesolimbic pathway. Unfortunately, the D2 receptors are simultaneously blocked throughout the brain in other pathways, such as the mesocortical, nigrostriatal, and tuberoinfundibular pathways. The mesocortical pathway is thought to be associated with negative symptoms. Therefore, blocking this pathway may induce secondary negative symptoms and cognitive effects. Occupying approximately 77% or more of the D2 receptors in the nigrostriatal pathway may increase the risk of extrapyramidal symptoms, such as dystonia (involuntary muscle contractions), akathisia (restlessness), bradykinesia (slow movements), and tardive dyskinesia. Chronic treatment with typical antipsychotics may result in 70–90% of D2 receptors being occupied [13]. It is estimated that about 5% of patients that maintain treatment with typical antipsychotics will develop tardive dyskinesia each year, making long-term therapy undesirable. A D2 blockade in the tuberoinfundibular pathway increases the risk for hyperprolactinemia, which may lead to more rapid demineralization of the bones, weight gain, and sexual dysfunction in both men and women. Several typical antipsychotics also block muscarinic M_1_ receptors, which may worsen cognitive blunting. Blocking the M_1_ receptor may also cause dry mouth, constipation, blurred vision, and urinary retention [14,15].

Second-generation (atypical) antipsychotics have a lower affinity for dopamine D2 receptors and greater affinities for other neuroreceptors, such as norepinephrine and serotonin receptors, especially at 5-HT_2A_. The risk for neurologic symptoms may be reduced with atypical antipsychotics, but the risk for metabolic problems, including weight gain, dyslipidemia, hypertension, and diabetes, has been observed to be higher, especially in patients treated with Clozapine or Olanzapine [13,16,17]. Antipsychotic medications, whether typical or atypical, can be toxic, potentially inducing any number of a lengthy list of neurologic, metabolic, and cardiovascular side effects that can ultimately contribute to a significantly decreased quality of life and reduced life expectancies [12,18,19,20,21,22]. Overall, typical antipsychotics often reduce the severity of positive symptoms, but are generally less effective at addressing negative symptoms [23,24,25]. Atypical antipsychotics are often assumed to be more efficacious for treating negative symptoms. However, intolerable side effects still lead to discontinuation of treatment [26,27,28]. The Clinical Antipsychotic Trials of Intervention Effectiveness (CATIE) failed to demonstrate that atypical antipsychotics were any more efficacious at treating negative psychotic symptoms than typical antipsychotics [16]. The Cost Utility of the Latest Antipsychotic Drugs in Schizophrenia Study (CUtLASS 1) failed to show a significant difference in the rates of treatment discontinuation, quality of life, or improvement in psychotic symptoms in comparing typical and atypical antipsychotics [29]. Common side effects of antipsychotics (e.g., constipation, weight gain, tardive dyskinesia, cardiovascular disturbances, and glucose metabolic dysregulation) can also contribute to the need for additional prescription medications for treating those side effects, resulting in added polypharmaceutical risks to patients [30,31,32].

Perhaps more fundamental to the drawbacks of the antipsychotic medication model for treating schizophrenia is the misapplication of the concept of “reductionism,” or the belief that complex mental illnesses, often characterized by unique mental symptoms, including mental intentions (e.g., obsessive beliefs, hypersensitivity to threatening stimuli, and low self-worth) can be reduced to biophysiological mechanisms, i.e., monoamine receptor sites, that function in basic or fundamental ways [33]. In fact, there is still little evidence and certainly no consensus on the innate biological (physiological or mental) functions of serotonergic, glutamatergic, or dopaminergic activity (up- or down-regulation) in their absolute and isolated forms, irrespective of the seemingly infinite factors, including past experiences and environmental conditions, associated with normative and anomalous mental states. Reductionist proposals of schizophrenia involve hyperactive dopaminergic signal transduction and the use of antidopaminergics as treatment, hyperactive glutamatergic signaling via NMDA receptors and the use of glutamatergics as treatment, and the role of muscarinic acetylcholine receptors and the use of positive allosteric modulators (PAMs) to indirectly regulate dopamine levels in areas of the brain involved in psychosis [34,35,36,37].

Another problem with antipsychotic pharmaceutical treatments is they are designed to act on particulate sites for treating the breadth of mental, behavioral, morphological and somatic symptoms associated with the diagnosis of schizophrenia [12,38]. The American Psychiatric Association’s criteria for a schizophrenic diagnosis includes only mental and behavioral symptoms: delusions, hallucinations, disorganized speech, grossly disorganized or catatonic behavior, and the presence of negative symptoms, which may include anhedonia, asociality, apathy, and alogia [39]. Two or more of the presentations must have existed for at least one month along with a few other criteria typically considered in a diagnosis, such as a major impairment in functioning for a significant period of time, signs of the disorder lasting for a continuous period of at least six months, and ruling out schizoaffective, bipolar, or depressive disorder with psychotic features.

However, schizophrenic symptomology also can occur in association with microglia activation and neuroinflammation [40,41,42,43], which increase permeability of the blood-brain barrier (BBB) [44]. This allows a wide range of both inorganic and organic toxins to disrupt neurological functioning, creating neuronal antibodies associated with schizophrenic symptomology [45,46,47]. Similar neuronal autoantibodies have been shown to arise as an immunological response to various cancers as well [48,49]. Non-paraneoplastic neural autoantibodies include those associated with hundreds of potential pathogens (e.g., rubella, influenza, *Varicella zoster*, *Candida albicans*, herpes, Lyme disease, *Toxoplasma gondii*, and several types of enteroviruses) that can lead to mental symptoms often described as “schizophrenic” (e.g., hallucinations, unusual involuntary movements) [50,51,52]. Additional sources of pathology leading to microglia activation and neuroinflammation may include food sensitivities, gastrointestinal inflammation, intestinal epithelial permeability, intestinal dysbiosis, nutrient deficiencies, environmental toxin exposure, and sleep deprivation, in addition to potential genetic sensitivities to express schizophrenic profiles [53,54,55,56,57].

Despite the association between schizophrenic symptomology and neuroinflammation, the DSM-5 focuses primarily on the mental and behavioral clusters of symptoms used in the diagnosis of schizophrenia without including physiological metrics and the many other types of schizophrenic symptoms not well treated with antipsychotics. Moreover, dozens of disorders have symptoms that overlap with schizophrenia, which are rarely evaluated prior to the diagnosis, likely due to the expense of more extensive biological and environmental tests, time, and the limited awareness of differential diagnoses that fall outside of a healthcare provider’s area of expertise. Patients diagnosed with schizophrenia often experience chronic immune system activation and high levels of pro-inflammatory cytokines, chemokines, and microglial activation [58,59]. Anti-inflammatory medications can alleviate symptoms associated with schizophrenia, and antipsychotic drugs are widely known to have anti-inflammatory and immunomodulatory effects [60]. It may be that a substantial percentage of the population of individuals prescribed antipsychotic medications for schizophrenia experience symptomatic relief primarily for these reasons as opposed to their actions on dopaminergic or serotonergic signaling pathways. Many cases exist in which patients originally diagnosed with schizophrenia were later found to have autoantibodies targeting the brain [61]. One of the more well-known autoantibodies targets the N-methyl-D-aspartate receptor. A recent study [62] among 121 patients with a schizophrenia diagnosis found evidence of NMDA-R antibodies in approximately 10% of the patients. While these autoantibodies may also be present in healthy populations, the prevalence was significantly higher in the population initially diagnosed with schizophrenia. Two of the patients from the study were later reclassified as having misdiagnosed NMDA-R encephalitis.

In light of the myriad psychophysiological characteristics that can accompany a schizophrenia diagnosis, perhaps greater emphasis should be directed toward interventions that operate on a systemic level rather than targeting isolated neurotransmitters. One treatment option that appears to have potential for regulating basic psychophysiological functioning is the *Cannabis* plant. Only relatively recently has the medical community begun to slowly warm to the idea that such historically high proportions of cannabis consumption among people with schizophrenia may reflect self-medication rather than recreational use of the plant [63,64]. Whereas cannabis was once often and sometimes still is described as a component cause of schizophrenia [65,66], several studies now suggest the use of medical cannabis as an effective therapy for schizophrenia [67,68]. According to the endocannabinoid deficiency theory, many mental and physical health disturbances result from a dysregulation of the body’s innate endocannabinoid system (ECS) [69,70,71,72], often described as a master network of chemical signals that promote somatic and psychological homeostasis or psychobiological state-efficiency [73,74,75]. The ECS consists of natural ligands (e.g., anandamide and 2-AG) and receptors (CB_1_ and CB_2_) that appear to play a major role in efficient regulation of systems that include sleep, feeding (e.g., gut permeability and adipogenesis), libido and fertility, pain perception, motivation, happiness, anxiety, learning and memory, social functioning, and cancer pathophysiology [70,76,77,78,79,80,81,82]. 

Many symptoms either directly associated with schizophrenia or that exacerbate psychosis might be alleviated by focusing on clinical endocannabinoid deficiencies. Interestingly, an elevation of anandamide levels in cerebrospinal fluid inversely correlates with psychotic symptoms [83,84]. One possibility is that anandamide may be released by the body in response to psychotic symptoms [85]. As such, *Cannabis* may be an effective and more tolerable treatment option for schizophrenia than conventional antipsychotic therapies because of its ability to regulate homeostasis via the ECS [84]. Studies involving experimental autoimmune encephalomyelitis (EAE) in mice have shown that during periods of CNS autoimmune inflammation, microglial cells become activated, proliferate, and localize to sites of inflammation [86,87]. CB_2_ receptors are mainly expressed in cells of the immune system [88] and become up-regulated on the microglial cells and other immune cells in the CNS during EAE [89,90]. Deletion of CB_1_ and CB_2_ receptors in animals has been shown to cause an exacerbated inflammatory phenotype in several models, due to an up-regulation of immune cell activity [91]. Notably, cannabidiol (CBD) treatment has been observed to act as an immunosuppressant and slow the progression of inflammation in animal and human studies [92,93,94]. Inflammation and oxidative stress are closely interconnected processes reinforcing each other [95]. In support of this notion, alterations in inflammatory, but also oxidative markers have been consistently detected in postmortem brain tissues, living patients, and translational animal studies [96,97,98]. Cellular redox homeostasis is modulated by the ECS [99], and cannabis administration in mice has been shown to modulate oxidative generation [100,101], a finding that led to the suggestion that CB_2_ receptors are potential target sites for Alzheimer’s disease [102,103].

In a recent placebo-controlled trial among schizophrenics [104], CBD treatment was shown to affect positive psychotic symptoms over and above the effect of a patient’s antipsychotic treatment. The researchers proposed several mechanisms of action within the ECS that may be responsible for the alleviation of positive psychotic symptoms: inhibition of fatty acid amide hydrolase (FAAH), inhibition of adenosine reuptake, TRPV1 and 5-HT_1A_ receptor agonism, and D2 high partial agonism. Further, the study indicated a favorable tolerability profile in the CBD group [104]. This is very meaningful given the many serious adverse effects of antipsychotics, effects long known to contribute to poorer health and wellbeing and reduced patient adherence.

Several studies have attempted to associate cannabis use with an increase in psychotic symptoms. It has been proposed that cannabis may influence N-methyl-D-aspartate receptors and cause NMDAR hypofunction [105]. A study from the University of Melbourne in Australia has also shown that distinctions in the cannabinoid system of the brain may be involved in the pathology of schizophrenia, including changes in CB_1_ receptors in the dorsolateral prefrontal cortex [106]. Another case study from the London Health Sciences Center concluded that a 38-year-old schizophrenic patient experienced a 20% decrease in striatal dopamine D2 receptor activity, suggesting that there was increased synaptic dopaminergic activity [107]. A major limitation in this study, and for other studies correlating cannabis consumption with schizophrenia, is that there was no mention of the cannabinoid profile within the strains of cannabis being used. These associations between cannabis use and the worsening of psychotic symptoms would appear to be primarily with tetrahydrocannabinol (Δ^9^-THC), the main psychoactive component in cannabis, which acts as a partial agonist at the CB_1_ and CB_2_ receptors. Indeed, there are data to support that THC exerts effects on the dopamine system and that it causes region-specific increases in dopamine release and nerve activity [108]. One possibility is that schizophrenic patients may tend to self-medicate with cannabis to treat negative symptoms and possibly overcome the effects of D2 blockade associated with antipsychotics. 

There are several studies indicating that CBD could block the temporary symptoms of psychosis exacerbated by THC. In one study, acute administration of THC modulated striatal and amygdala activation and its effects correlated with psychotic and anxiety symptoms, but CBD had an opposite effect on neural activation in these regions, adding to an already robust body of evidence supporting the hypothesis that combined administration of CBD and THC result in reduced paranoia [109]. In another study among 88 patients diagnosed with schizophrenia, patients were randomized to receive either CBD or a placebo alongside their existing antipsychotic medication. After six weeks of treatment, compared to the placebo group, the CBD group had lower levels of positive psychotic symptoms and greater improvements in cognitive performance. The CBD was well tolerated, and rates of adverse events were similar between the two groups, suggesting that CBD may be a useful adjunctive therapy in schizophrenia, especially considering that its mechanism of action does not depend upon dopamine receptor antagonism [104]. Because CBD has no significant affinity at CB_1_ and CB_2_ receptors, it is generally believed that CBD may function as a non-competitive negative allosteric modulator of the CB_1_ receptor [110] and as an indirect antagonist of CB_1_ and CB_2_ receptors more generally [93,111]. Proposed mechanisms for how CBD may reduce inflammation associated with psychosis include: moderately blocking FAAH, thereby inhibiting breakdown of anandamide; breaking down other ethanolamides that are a part of the endocannabinoid system, such as palmitoylethanolamide (PEA) and docosatetraenoylethanolamide (DEA), and blocking anandamide transporters that compete with fatty acid-binding proteins (FABPs) [112,113,114]. Unfortunately, due to cannabis’ continued Schedule I status and associated barriers to conducting medical cannabis research [115], no practical, naturalistic investigations have been completed on how patient-managed phytocannabinoid consumption immediately affects schizophrenic symptoms in real-time.

In conclusion, sustained states of inflammation in the gut and brain may be caused by genetic, neurological, autoimmune, endocrine, oncological, pharmacological, nutritional, and other environmental factors, including stress, sleep deprivation, heavy metal toxicity, phasic and chronic infection(s), intestinal dysbiosis, and low-grade sensitivities to foods, pollutants, teratogens, and chemicals [2,116]. Given the association between inflammation, microglial activation, and schizophrenic symptomology, it may be that a significant number of cases of schizophrenia arise from autoantibodies targeting the CNS [61,62]. While a variety of triggers likely contribute to psychosis, inflammation and CNS immune system activation are almost always present. A review of the literature suggests that CBD in particular may be a safe and effective treatment option for schizophrenia as a primary or adjunctive therapy, supporting both inflammatory causes of schizophrenia and the potential importance of targeting the ECS in treating this poorly understood disease rather than ill-tolerated antipsychotics with debilitating side effects.

## References

[1] Bartels S.J., Clark R.E., Peacock W.J., Dums A.R., Pratt S.I. (2003). Medicare and Medicaid costs for schizophrenia patients by age cohort compared with costs for depression, dementia, and medically ill patients. Am. J. Geriatr. Psychiatry.

[2] Messias E., Chen C.-Y., Eaton W.W. (2007). Epidemiology of schizophrenia: Review of findings and myths. Psychiatr. Clin. N. Am..

[3] Saha S., Chant D., Welham J., McGrath J. (2005). A Systematic Review of the Prevalence of Schizophrenia. PLoS Med..

[4] Desai P.R., Lawson K.A., Barner J.C., Rascati K.L. (2013). Estimating the direct and indirect costs for community-dwelling patients with schizophrenia. J. Pharm. Health Serv. Res..

[5] Alam R., Abdolmaleky H.M., Zhou J.-R. (2017). Microbiome, inflammation, epigenetic alterations, and mental diseases. Am. J. Med. Genet. Part B.

[6] Brown A.S., Lau F.S., Pletnikov M.V., Waddington J.L. (2016). A review of the epidemiology of schizophrenia. Modeling the Psychopathological Dimensions of Schizophrenia: From molecules to behavior.

[7] Roth T.L., Lubin F.D., Sodhi M., Kleinman J.E. (2009). Epigenetic mechanisms in schizophrenia. Biochim. Biophys. Acta.

[8] Buchanan R.W., Weiner E., Kelly D.L., Gold J.M., Keller W.R., Waltz J.A., McMahon R.P., Gorelick D.A. (2015). Rasagiline in the Treatment of the Persistent Negative Symptoms of Schizophrenia. Schizophr. Bull..

[9] Watanabe K., Miura I., Kanno-Nozaki K., Horikoshi S., Mashiko H., Niwa S., Yabe H. (2015). Associations between five-factor model of the Positive and Negative Syndrome Scale and plasma levels of monoamine metabolite in patients with schizophrenia. Psychiatry Res..

[10] Delgado P.L. (2000). Depression: The case for a monoamine deficiency. J. Clin. Psychiatry.

[11] Hirvonen J., Hietala J., Seeman P., Madras B. (2014). Dopamine receptor imaging in schizophrenia: Focus on genetic vulnerability. Imaging of the Human Brain in Health and Disease.

[12] Ng J., Papandreou A., Heales S., Kurian M. (2015). Monoamine neurotransmitter disorders—Clinical advances and future perspectives. Nat. Rev. Neurology.

[13] Tahir R. (2007). Metabolic Effects of Atypical Antipsychotics. https://www.uspharmacist.com/article/metabolic-effects-of-atypical-antipsychotics.

[14] Guzmán F. (2016). First-generation Antipsychotics: An Introduction. https://psychopharmacologyinstitute.com/antipsychotics/first-generation-antipsychotics/.

[15] Stahl S.M. (2008). Stahl’s Essential Psychopharmacology: Neuroscientific Basis and Practical Applications.

[16] Lieberman J.A., Stroup T.S., McEvoy J.P., Swartz M.S., Rosenheck R.A., Perkins D.O., Keefe R.S.E., Davis S.M., Davis C.E., Lebowitz B.D. (2005). Effectiveness of antipsychotic drugs in patients with chronic schizophrenia. N. Engl. J. Med..

[17] Rodriguez A.P., Tajima-Pozo K., Lewczuk A., Montañes-Rada F. (2015). Atypical antipsychotics and metabolic syndrome. Cardiovasc. Endocrinol..

[18] Browne S., Roe M., Lane A., Gervin M., Dooher M., Kinsella A., Larkin C., O’Callaghan E. (1996). Quality of life in schizophrenia: Relationship to sociodemographic factors, symptomatology and tardive dyskinesia. Acta Psychiatr. Scand..

[19] Chong S., Tay J.A., Subramaniam M., Pek E., Machin D. (2009). Mortality rates among patients with schizophrenia and tardive dyskinesia. J. Clin. Psychopharmacol..

[20] Dean C., Thuras P. (2009). Mortality and tardive dyskinesia: Long-term study using the US National Death Index. Br. J. Psychiatry.

[21] Minns A.B., Clark R.F. (2012). Toxicology and overdose of atypical antipsychotics. J. Emerg. Med..

[22] West S., Rowbotham D., Xiong G., Kenedi C. (2017). Clozapine induced gastrointestinal hypomotility: A potentially life threatening adverse event. A review of the literature. Gen. Hosp. Psychiatry.

[23] Correll C.U., Rubio J.M., Inczedy-Farkas G., Birnbaum M.L., Kane J.M., Leucht S. (2017). Efficacy of 42 pharmacologic cotreatment strategies added to antipsychotic monotherapy in schizophrenia. Systematic overview and quality appraisal of the meta-analytic evidence. JAMA Psychiatry.

[24] Goff D.C., Falkai P., Fleischhacker W.W., Girgis R.R., Kahn R.M., Uchida H., Zhao J., Lieberman J.A. (2017). The long-term effects of antipsychotic medication on clinical course in schizophrenia. Am. J. Psychiatry.

[25] Leucht S., Corves C., Arbter D., Engel R.R., Li C., Davis J.M. (2008). Second-generation versus first-generation antipsychotic drugs for schizophrenia: A meta-analysis. Lancet.

[26] Andrade C.C. (2016). Antipsychotic drugs in schizophrenia: Relative effects in patients with and without treatment resistance. J. Clin. Psychiatry.

[27] Lieberman J. (2005). Effectiveness of Antipsychotic Drugs in Patients with Chronic Schizophrenia. NEJM.

[28] Tonin F.S., Piazza T., Wiens A., Fernandez-Llimos F., Pontarolo R. (2015). Adverse events and treatment failure leading to discontinuation of recently approved antipsychotic drugs in schizophrenia: A network meta-analysis. Schizophr. Res..

[29] Jones P.B., Barnes T.R.E., Davies L., Dunn G., Lloyd H., Hayhurst K.P., Murray R.M., Markwick A., Lewis S.W. (2006). Randomized controlled trial of the effect on quality of life of second- vs first-generation antipsychotic drugs in schizophrenia. Arch. Gen. Psychiatry.

[30] Correll C.U., Kane J.M., Citrome L. (2017). Epidemiology, Prevention, and Assessment of Tardive Dyskinesia and Advances in Treatment: (Academic Highlights). J. Clin. Psychiatry.

[31] Takeuchi H., Suzuki T., Remington G., Uchida H. (2015). Antipsychotic polypharmacy and corrected QT interval: A systematic review. Can. J. Psychiatry.

[32] Zhang Y., Liu Y., Su Y., You Y., Ma Y., Yang G., Song Y., Liu X., Wang M., Zhang L. (2017). The metabolic side effects of 12 antipsychotic drugs used for the treatment of schizophrenia on glucose: A network meta-analysis. BMC Psychiatry.

[33] Borsboom D., Cramer A., Kalis A. (2018). Brain disorders? Not really… Why network structures block reductionism in psychopathology research. Behav. Brain Sci..

[34] Boyd K.N., Mailman R.B. (2012). Dopamine receptor signaling and current and future antipsychotic drugs. Handb. Exp. Pharmacol..

[35] Foster D.J., Jones C.K., Conn P.J. (2012). Emerging approaches for treatment of schizophrenia: Modulation of cholinergic signaling. Discov. Med..

[36] Melancon B.J., Tarr J.C., Panarese J.D., Wood M.R., Lindsley C.W. (2013). Allosteric modulation of the M_1_ muscarinic acetylcholine receptor: Improving cognition and a potential treatment for schizophrenia and Alzheimer’s disease. Drug Discov. Today.

[37] Menniti F.S., Lindsley C.W., Conn P.J., Pandit J., Zagouras P., Volkmann R.A. (2013). Allosteric modulators for the treatment of schizophrenia: Targeting glutamatergic networks. Curr. Top. Med. Chem..

[38] Suddath R.L., Christison G.W., Torrey E.F., Casanova M., Weinberger D.R. (1990). Anatomical abnormalities in the brains of monozygotic twins discordant for schizophrenia. N. Engl. J. Med..

[39] American Psychiatric Association (2013). Diagnostic and Statistical Manual of Mental Disorders.

[40] Monji A., Kato T.A., Mizoguchi Y., Horikawa H., Seki Y., Kasai M., Yamada S., Kanba S. (2013). Neuroinflammation in schizophrenia especially focused on the role of microglia. Progr. Neuropsychopharmacol. Biol. Psychiatry.

[41] Aricioglu F., Ozkartal C.S., Unal G., Dursun S., Cetin M., Mueller N. (2016). Neuroinflammation in schizophrenia: A critical review and the future. Bull. Clin. Psychopharmacol..

[42] Müller N., Weidinger E., Leitner B., Schwarz M.J. (2015). The role of inflammation in schizophrenia. Front. Neurosci..

[43] Trépanier M.O., Hopperton K.E., Mizrahi R., Mechawar N., Bazinet R.P. (2016). Postmortem evidence of cerebral inflammation in schizophrenia: A systematic review. Mol. Psychiatry.

[44] Da Fonseca A.C.C., Matias D., Garcia C., Amaral R., Geraldo L.H., Freitas C., Lima F.R.S. (2014). The impact of microglial activation on blood-brain barrier in brain diseases. Front. Cell Neurosci..

[45] Schoknecht K., Shalev H. (2012). Blood–brain barrier dysfunction in brain diseases: Clinical experience. Epilepsia.

[46] Steiner J., Bogerts B., Sarnyai Z., Walter M., Gos T., Bernstein H.G., Myint A.M. (2012). Bridging the gap between the immune and glutamate hypotheses of schizophrenia and major depression: Potential role of glial NMDA receptor modulators and impaired blood–brain barrier integrity. World J. Biol. Psychiatry.

[47] Stolp H.B., Dziegielewska K.M., Ek C.J., Potter A.M., Saunders N.R. (2005). Long-term changes in blood–brain barrier permeability and white matter following prolonged systemic inflammation in early development in the rat. Eur. J. Neurosci..

[48] Banjara M., Ghosh C., Dadas A., Mazzone P., Janigro D. (2017). Detection of brain-directed autoantibodies in the serum of non-small cell lung cancer patients. PLoS ONE.

[49] Finke C., Bartels F., Lütt A., Prüss H., Harms L. (2017). High prevalence of neuronal surface autoantibodies associated with cognitive deficits in cancer patients. J. Neurol..

[50] Abboud H., Rossman I., Mealy M., Hill E., Thompson N., Banerjee A., Probasco J., Levy M. (2017). Neuronal autoantibodies: Differentiating clinically relevant and clinically irrelevant results. J. Neurol..

[51] Brown A.S. (2006). Prenatal infection as a risk factor for schizophrenia. Schizophr. Bull..

[52] Lang K., Prüss H. (2017). Frequencies of neuronal autoantibodies in healthy controls: Estimation of disease specificity. Neurol. Neuroimmunol. Neuroinflammation.

[53] Arinola G., Idonije B., Akinlade K., Ihenyen O. (2010). Essential trace metals and heavy metals in newly diagnosed schizophrenic patients and those on anti-psychotic medication. J. Res. Med. Sci. Off. J. Isfahan Univ. Med. Sci..

[54] Dean K., Murray R.M. (2005). Environmental risk factors for psychosis. Dialogues Clin. Neurosci..

[55] Howes O.D., McCutcheon R. (2017). Inflammation and the neural diathesis-stress hypothesis of schizophrenia: A reconceptualization. Transl. Psychiatry.

[56] Petrovsky N., Ettinger U., Hill A., Frenzel L., Meyhöfer I., Wagner M., Kumari V. (2014). Sleep deprivation disrupts prepulse inhibition and induces psychosis-like symptoms in healthy humans. J. Neurosci..

[57] Severance E.G., Prandovszky E., Castiglione J., Yolken R.H. (2015). Gastroenterology issues in schizophrenia: Why the gut matters. Curr. Psychiatry Rep..

[58] Beumer W., Drexhage R.C., De Wit H., Versnel M.A., Drexhage H.A., Cohen D. (2012). Increased level of serum cytokines, chemokines and adipokines in patients with schizophrenia is associated with disease and metabolic syndrome. Psychoneuroendocrinology.

[59] Reale M., Patruno A., De Lutiis M.A., Pesce M., Felaco M., Di Giannantonio M., Di Nicola M., Grilli A. (2011). Dysregulation of chemo-cytokine production in schizophrenic patients versus healthy controls. BMC Neurosci..

[60] Müller N., Weidinger E., Leitner B., Schwarz M. (2016). The role of inflammation and the immune system in schizophrenia. Neurobiol. Schizophr..

[61] Riedmüller R., Müller S. (2017). Ethical Implications of the Mild Encephalitis Hypothesis of Schizophrenia. Front. Psychiatry.

[62] Steiner J., Walter M., Glanz W., Sarnyai Z., Bernstein H.-G., Vielhaber S., Kästner A., Skalej M., Jordan W., Schiltz K. (2013). Increased Prevalence of Diverse *N*-Methyl-d-Aspartate Glutamate Receptor Antibodies in Patients with an Initial Diagnosis of Schizophrenia Specific Relevance of IgG NR1a Antibodies for Distinction from *N*-Methyl-d-Aspartate Glutamate Receptor Encephalitis. JAMA Psychiatry.

[63] National Academies of Sciences, Engineering, and Medicine, Health and Medicine Division, Board on Population Health and Public Health Practice, Committee on the Health Effects of Marijuana (2017). An Evidence Review and Research Agenda.

[64] Proal A.C., Fleming J., Galvez-Buccollini J.A., DeLisi L.E. (2014). A controlled family study of cannabis users with and without psychosis. Schizophr. Res..

[65] Di Forti M., Marconi A., Carra E., Fraietta S., Trotta A., Bonomo M., Bianconi F., Gardner-Sood P., O’Connor J., Russo M. (2015). Proportion of patients in south London with first-episode psychosis attributable to use of high potency cannabis: A case-control study. Lancet Psychiatry.

[66] Gage S.H., Jones H.J., Burgess S., Bowden J., Davey Smith G., Zammit S., Munafo M.R. (2017). Assessing causality in associations between cannabis use and schizophrenia risk: A two-sample Mendelian randomization study. Psychol. Med..

[67] Fakhoury M. (2016). Could cannabidiol be used as an alternative to antipsychotics?. J. Psychiatr. Res..

[68] Gururajan A., Malone D.T. (2016). Does cannabidiol have a role in the treatment of schizophrenia?. Schizophr. Res..

[69] Di Marzo V., Stella N., Zimmer A. (2015). Endocannabinoid signaling and the deteriorating brain. Nat. Rev. Neurosci..

[70] Karhson D.S., Hardan A.Y., Parker K.J. (2016). Endocannabinoid signaling in social functioning: An RDoC perspective. Transl. Psychiatry.

[71] Russo E. (2018). Clinical Endocannabinoid Deficiency (CECD): Can this concept explain therapeutic benefits of cannabis in migraine, fibromyalgia, irritable bowel syndrome and other treatment-resistant conditions?. Neuro Endocrinol. Lett..

[72] Smith S.C., Wagner M.S. (2014). Clinical endocannabinoid deficiency (CECD) revisited: Can this concept explain the therapeutic benefits of cannabis in migraine, fibromyalgia, irritable bowel syndrome and other treatment-resistant conditions?. Neuro Endocrinol. Lett..

[73] Acharya N., Penukonda S., Shcheglova T., Hagymasi A.T., Basu S., Srivastava P.K. (2017). Endocannabinoid system acts as a regulator of immune homeostasis in the gut. Proc. Natl. Acad. Sci. USA.

[74] Bermudez-Silva F.J., Viveros M.P., McPartland J.M., Rodriguez de Fonseca F. (2010). The endocannabinoid system, eating behavior and energy homeostasis: The end or a new beginning?. Pharmacol. Biochem. Behav..

[75] Silvestri C., Di Marzo V. (2013). The endocannabinoid system in energy homeostasis and the etiopathology of metabolic disorders. Cell Metab..

[76] Androvicova R., Horace J., Stark T., Drago F., Micale V. (2017). Endocannabinoid system in sexual motivational processes: Is it a novel therapeutic horizon?. Pharmacol. Res..

[77] Cani P.D. (2012). Crosstalk between the gut microbiota and the endocannabinoid system: Impact on the gut barrier function and the adipose tissue. Clin. Microbiol. Infect..

[78] Du Plessis S.S., Agarwal A., Syriac A. (2015). Marijuana, phytocannabinoids, the endocannabinoid system, and male fertility. J. Assist. Reprod. Genet..

[79] Muccioli G.G., Naslain D., Bäckhed F., Reigstad C.S., Lambert D.M., Delzenne N.M., Cani P.D. (2010). The endocannabinoid system links gut microbiota to adipogenesis. Mol. Syst. Biol..

[80] Pava M.J., Makriyannis A., Lovinger D.M. (2016). Endocannabinoid signaling regulates sleep stability. PLoS ONE.

[81] Sierra S., Luquin N., Navarro-Otano J. (2018). The endocannabinoid system in cardiovascular function: Novel insights and clinical implications. Clin. Auton. Res..

[82] Tegeder I. (2016). Endocannabinoids as guardians of metastasis. Int. J. Mol. Sci..

[83] Morgan C., Page E., Schaefer C., Chatten K., Manocha A., Gulati S., Brandner B., Leweke F. (2013). Cerebrospinal fluid anandamide levels, cannabis use and psychotic-like symptoms. Br. J. Psychiatry.

[84] Giuffrida A., Leweke F.M., Gerth C., Schreiber D., Koethe D., Faulhaber J., Klosterkötter J., Piomelli D. (2004). Cerebrospinal Anandamide Levels are Elevated in Acute Schizophrenia and are Inversely Correlated with Psychotic Symptoms. Neuropsychopharmacology.

[85] Leweke F.M., Piomelli D., Pahlisch F., Muhl D., Gerth C.W., Hoyer C., Klosterkötter J., Hellmich M., Koethem D. (2012). Cannabidiol enhances anandamide signaling and alleviates psychotic symptoms of schizophrenia. Transl. Psychiatry.

[86] Graeber M.B., Li W., Rodriguez M.L. (2011). Role of microglia in CNS inflammation. FEBS Lett..

[87] Kim Y., Joh T.H. (2006). Microglia, major player in the brain inflammation: Their roles in the pathogenesis of Parkinson’s disease. Exp. Mol. Med..

[88] Galiègue S., Mary S., Marchand J., Dussossoy D., Carrière D., Carayon P., Bouaboula M., Shire D., Fur G., Casellas P. (1995). Expression of central and peripheral cannabinoid receptors in human immune tissues and leukocyte subpopulations. Eur. J. Biochem..

[89] Maresz K., Carrier E.J., Ponomarev E.D., Hillard C.J., Dittel B.N. (2005). Modulation of the cannabinoid CB_2_ receptor in microglial cells in response to inflammatory stimuli. J. Neurochem..

[90] Cabral G.A., Griffin-Thomas L. (2009). Emerging role of the CB_2_ cannabinoid receptor in immune regulation and therapeutic prospects. Expert Rev. Mol. Med..

[91] Karmaus P.W.F., Chen W., Crawford R.B., Harkema J.R., Kaplan B.L.F., Kaminski N.E. (2011). Deletion of cannabinoid receptors 1 and 2 exacerbates APC function to increase inflammation and cellular immunity during influenza infection. J. Leukoc. Biol..

[92] Burstein S. (2015). Cannabidiol (CBD) and its analogs: A review of their effects on inflammation. Bioorg. Med. Chem..

[93] McPartland J.M., Duncan M., Di Marzo V., Pertwee R.G. (2015). Are cannabidiol and Δ^9^-tetrahydrocannabivarin negative modulators of the endocannabinoid system? A systematic review. Br. J. Pharmacol..

[94] Turcotte C., Blanchet M., Laviolette M., Flamand N. (2016). The CB_2_ receptor and its role as a regulator of inflammation. Cell. Mol. Life Sci..

[95] Biswas S. (2016). Does the Interdependence between oxidative stress and inflammation explain the antioxidant paradox?. Oxid. Med. Cell. Longev..

[96] Emiliani F.E., Sedlak T.W., Sawa A. (2014). Oxidative stress and schizophrenia: Recent breakthroughs from an old story. Curr. Opin. Psychiatry.

[97] Gonzalez-Liencres C., Tas C., Brown E.C., Erdin S., Onur E., Cubukcoglu Z., Aydemir O., Esen-Danaci A., Brüne M. (2014). Oxidative stress in schizophrenia: A case-control study on the effects on social cognition and neurocognition. BMC Psychiatry.

[98] Schiavone S., Jaquet V., Trabace L., Krause K.-H. (2013). Severe life stress and oxidative stress in the brain: From animal models to human pathology. Antioxid. Redox Signal..

[99] Lipina C., Hundal H.S. (2016). Modulation of cellular redox homeostasis by the endocannabinoid system. Open Biol..

[100] Abdel-Salam O.M.E., El-Shamarka M.E.-S., Salem N.A., El-Din MGaafar A. (2012). Effects of Cannabis sativa extract on haloperidol-induced catalepsy and oxidative stress in the mice. EXCLI J..

[101] Valvassori S.S., Elias G., de Souza B., Petronilho F., Dal-Pizzol F., Kapczinski F., Trzesniak C., Tumas V., Dursun S., Nisihara Chagas M.H. (2009). Effects of cannabidiol on amphetamine-induced oxidative stress generation in an animal model of mania. J. Psychopharmacol..

[102] Aso E., Ferrer I. (2016). CB_2_ Cannabinoid Receptor ss Potential Target against Alzheimer’s Disease. Front. Neurosci..

[103] Koppel J., Davies P. (2008). Targeting the endocannabinoid system in Alzheimer’s disease. J. Alzheimer’s Disease.

[104] Mcguire P., Robson P., Cubala W.J., Vasile D., Morrison P.D., Barron R., Taylor A., Wright S. (2017). Cannabidiol (CBD) as an adjunctive therapy in schizophrenia: A multicenter randomized controlled trial. Am. J. Psychiatry.

[105] Sanchez Blazquez P., Rodriguez-Munoz M., Sanchez-Blazquez P., Rodriguez-Munoz M., Garzon J. (2014). The cannabinoid receptor 1 associates with NMDA receptors to produce glutamatergic hypofunction: Implications in psychosis and schizophrenia. Front. Pharmacol..

[106] Dean B., Sundram S., Bradbury R., Scarr E., Copolov D. (2001). Studies on [3H]CP-55940 binding in the human central nervous system: Regional specific changes in density of cannabinoid-1 receptors associated with schizophrenia and cannabis use. Neuroscience.

[107] Voruganti L.N., Slomka P., Zabel P., Mattar A., Awad A.G. (2001). Case report: Cannabis induced dopamine release: An in-vivo SPECT study. Psychiatry Res. Neuroimaging.

[108] Bloomfield M.A., Ashok A.H., Volkow N.D., Howes O.D. (2016). The effects of Δ9-tetrahydrocannabinol on the dopamine system. Nature.

[109] Bhattacharyya S., Morrison P.D., Fusar-Poli P., Martín-Santos R., Borgwardt S.J., Winton-Brown T., Nosarti C., O’Carroll C.M., Seal M., Allen P. (2010). Opposite effects of Δ-9-tetrahydrocannabinol and cannabidiol on human brain function and psychopathology. Neuropsychopharmacology.

[110] Laprairie R.B., Bagher A.M., Kelly M.E.M., Denovan-Wright E.M. (2015). Cannabidiol is a negative allosteric modulator of the cannabinoid CB_1_ receptor. Br. J. Pharmacol..

[111] Pertwee R. (2008). The diverse CB_1_ and CB_2_ receptor pharmacology of three plant cannabinoids: Δ9-tetrahydrocannabinol, cannabidiol and Δ9-tetrahydrocannabivarin. Br. J. Pharmacol..

[112] Rohleder C., Müller J.K., Lange B., Leweke F.M. (2016). Cannabidiol as a potential new type of an antipsychotic. A critical review of the evidence. Front. Pharmacol..

[113] Alhouayek M., Bottemanne P., Muccioli G.G., Makriyannis A. (2017). *N*-acylethanolamine-hydrolyzing acid amidase and fatty acid amide hydrolase inhibition differentially affect *N-*acylethanolamine levels and macrophage activation. Biochim. Biophys. Acta-Mol. Cell Biol. Lipids.

[114] Alhouayek M., Muccioli G.G. (2014). Harnessing the anti-inflammatory potential of palmitoylethanolamide. Drug Discov. Today.

[115] Stith S.S., Vigil J.M.V. (2016). Federal barriers to *Cannabis* research. Science.

[116] Griswold K.S., Regno P.A., Berger R.C. (2015). Recognition and Differential Diagnosis of Psychosis in Primary Care. Am. Fam. Phys..

